# The SOX12-YBX1-LDHA signaling axis drives metastasis in papillary thyroid carcinoma

**DOI:** 10.1038/s41419-025-07797-5

**Published:** 2025-07-01

**Authors:** Xianhui Ruan, Yue Huang, Yu Zeng, Zhenhao Zhao, Mei Tao, Zewei Zhao, Yuqi Wang, Guangwei Xu, Wei Zhang, Jialong Yu, Wei Luo, Songfeng Wei, Xichuan Li, Ming Gao, Yang Yu, Peng Li, Xiangqian Zheng

**Affiliations:** 1 Department of Thyroid and Neck Tumor, Tianjin Medical University Cancer Institute and Hospital, National Clinical Research Center for Cancer, Key Laboratory of Cancer Prevention and Therapy, Tianjin’s Clinical Research Center for Cancer, Tianjin, 300060 China; 2 College of Life Sciences, State Key Laboratory of Medicinal Chemical Biology, Frontiers Science Center for Cell Responses, Nankai University, Tianjin, China; 3 Department of Thyroid and Breast Surgery, Tianjin Key Laboratory of General Surgery in Construction, Tianjin Union Medical Center, Tianjin, 300121 China; 4 School of Medicine, Nankai University, Tianjin, China; 5 Tianjin Key Laboratory of Animal and Plant Resistance, College of Life Sciences, Tianjin Normal University, Tianjin, China

**Keywords:** Head and neck cancer, Head and neck cancer, Cancer genetics

## Abstract

The sex-determining region Y (SRY)-box (SOX) family plays crucial roles in carcinogenesis and cancer progression. However, the precise function of SOX12 in papillary thyroid carcinoma (PTC) metastasis remains to be investigated. In this study, we analyzed single-cell and bulk RNA sequencing (RNA-seq) datasets and demonstrated significant upregulation of SOX12 in PTC, which is associated with poor prognosis in PTC patients. Functional assays demonstrated that SOX12 overexpression promoted the metastasis of PTC cells, whereas the downregulation of SOX12 markedly reduced the aggressiveness of PTC. By integrating RNA-seq, CUT&Tag, and immunoprecipitation mass spectrometry (IP-MS), we found that SOX12 directly upregulated YBX1 expression and recruited it to the *LDHA* promoter, thus leading to activation of the TGF-β signaling pathway. Crucially, *LDHA* knockdown rescued SOX12/YBX1-mediated TGF-β signaling activation and inhibited the migration and invasion of PTC cells. Furthermore, we demonstrated that SOX12 expression is positively correlated with YBX1 and LDHA expression levels in clinical PTC samples. Taken together, these results reveal a critical link between the SOX12-YBX1-LDHA signaling axis and PTC metastasis and suggest that targeting of this signaling node may be a promising alternative therapeutic strategy to combat PTC metastasis.

## Introduction

Papillary thyroid cancer (PTC) is generally indolent, with good outcomes and long-term survival. However, PTC distant metastasis is often a dangerous event and accounts for most of its disease-specific mortality [1, 2]. Compared with the mortality rate of nonmetastatic patients (4.3%), metastatic patients have an increased mortality rate (55.5%). Moreover, multiple organ metastasis is associated with a higher mortality rate (50.4%) than single organ involvement (36.7%) [3, 4]. The limited understanding of the molecular mechanisms underlying PTC invasion and metastasis limits efforts to develop effective therapies. Thus, the identification of novel molecular drivers with therapeutic potential for advanced or metastatic PTC is urgently needed.

SOX12 is a well-known oncogene and a member of the SOX C family of SRY-related HMG-box (SOX) transcription factors [5]. The increased expression of SOX12 has been reported to be associated with malignant transformation and metastasis in several cancer types, including colorectal, lung, liver, gastric, and breast cancers [6–11]. Du et al. reported that SOX12 enhances gastric cell metastasis via direct binding to the MMP7 and IGF1 promoters, thus upregulating their expression [7]. Huang et al. reported that overexpression of SOX12 induced epithelial-mesenchymal transition and promoted liver cancer metastasis by transactivating Twist1 and FGFBP1 expression [8]. In the context of thyroid cancer, SOX12 was reported to promote thyroid cancer invasion through interactions with the POU family [9]. However, as a transcription factor, the epigenetic regulatory role of SOX12 in thyroid cancer progression has not yet been reported.

As a member of the RBP family, YBX1 contains an evolutionarily conserved cold-shock domain (CSD). Nuclear YBX1 can specifically bind to the promoters of targeted genes to regulate their transcription and translation, whereas in the cytoplasm, it forms complexes with messenger ribonucleoproteins (mRNPs) and regulates mRNA stability [12, 13]. Due to its numerous cellular functions, YBX1 has been demonstrated to be involved in various human malignancies, including pancreatic cancer, breast cancer, lung cancer, multiple myeloma, osteosarcoma, synovial sarcoma, prostate cancer, and ovarian cancer [14]. YBX1 drives glycolysis and EMT in triple-negative breast cancer [15]. Additionally, YBX1 and LDHA are coregulated hub genes in a hypoxia-glycolysis-lactate axis linked to HCC prognosis [16]. However, the role of YBX1 in PTC remains largely unknown.

Here, we show that SOX12 overexpression is correlated with PTC metastasis and suggests a poor prognosis. In addition, SOX12 drives PTC metastasis via the regulation of LDHA protein, which was identified as an important metastasis-promoting gene of PTC in our previous study [17]. Moreover, we demonstrated that SOX12 directly transcriptionally activates *YBX1* and cooperates with YBX1 to form a functional immunocomplex on the LDHA promoter to promote LDHA transcriptional activity in PTC cells. Our findings may lead to a novel strategy to interrupt the SOX12-YBX1-LDHA signaling pathway within the metastatic cascade to treat this disease.

## Results

### Single-cell RNA-seq analysis identifies SOX12 as a metastasis-associated gene for PTC

**Fig. 1:**
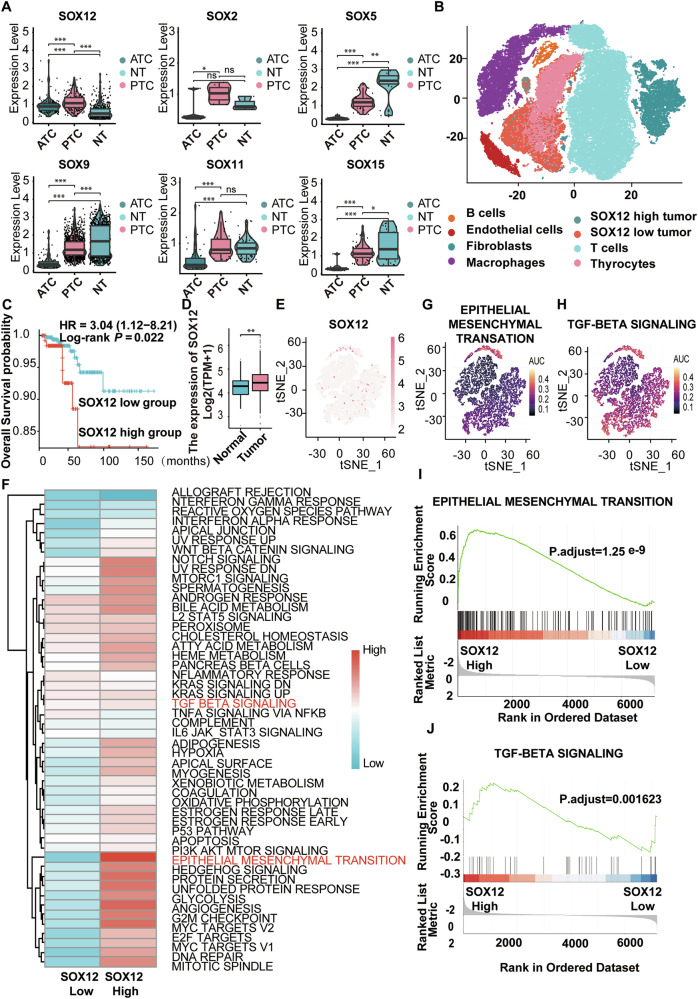
Signal cell RNA-seq analysis identifies SOX12 as a metastasis associated gene for PTC. **A** Expression of 6 representative genes of SOX family in thyroid scRNA-seq data, data are shown as the median with a range. **B** A t-SNE plot displaying the clustering of single-cell data from 10 samples into 6 distinct groups. **C** Kaplan–Meier plot of overall survival (OS) of patients with TC from TCGA STAD and GTEX cohort. **D** Expression of SOX12 between tumor and normal cells in TCGA STAD and GTEX cohort. **E** t-SNE plots of SOX12 expression in tumor subgroups. **F** Heatmap of differently activated pathways between SOX12 high tumors and SOX12 low tumors using GSVA algorithm. **G**, **H** epithelial mesenchymal transformation pathway, and TGF-beta signaling pathway in thyroid cell subgroups using AUCell (**G**, **H**). **I**, **J** GSEA plots verified the two significantly enriched pathways (epithelial mesenchymal transition and TGF-beta signaling) in the SOX12 high tumors compared to the SOX12 low tumors. *P* values were determined using a two-tailed unpaired Student’s *t* test. (*p < 0.05, **p < 0.01, ***p < 0.001).

We integrated several thyroid cancer single-cell sequencing (scRNA-seq) datasets to investigate the importance of SOX12 transcripts during thyroid cancer (TC) progression (GSE148673, GSE184362, and GSE134355). The expression of SOX12 was higher in the tumor cell subgroup compared to normal subgroup, whereas other SOX transcripts, including SOX2, SOX5, SOX9, SOX11, and SOX15, had low expression in tumor clusters (Fig. 1A, E). The identification of 8 major cell types, including T/NK cells, B cells, myeloid cells, fibroblasts, endothelial cells, SOX12-high tumors, SOX12-low tumors, and normal thyroid cells, was accomplished by integrating singleR automated prediction with manual prediction via established biomarkers which we have reported before [18] (Fig. 1B, Supplementary Fig. S1A–E). We then divided the epithelial cell subsets into anaplastic thyroid carcinoma (ATC), PTC, and normal cell subsets via the TDS scores which we have reported before (Supplementary Fig. S1F). In the TCGA-STAD and GTEX cohorts, the expression of SOX12 was significantly associated with overall survival (Fig. 1C) and higher in tumor tissues than in normal tissues or tumor-adjacent normal tissues across different cancer types (Fig. 1D). We used the gene set variation analysis (GSVA) algorithm in the scRNA-seq data to compare the enrichment pathways that differed from those associated with SOX12 expression to better understand the biological significance of SOX12 in the PTC subgroup. We discovered that the epithelial-mesenchymal transformation (EMT) pathway was the most highly enriched pathway (Fig. 1F). Since TGF-β signaling pathways are essential for the aggressiveness of thyroid cancer via EMT, t-SNE plots of both the EMT and TGF-β signaling pathways in thyroid cell subgroups were generated via the AUCell algorithm (Fig. 1G, H). The volcano plot revealed that most genes were upregulated in the scRNA-seq data (Supplementary Fig. S1G). Furthermore, we used KEGG enrichment of the differentially expressed genes (DEGs), which are significantly enriched in TGF-β signaling pathways (Supplementary Fig. S1H). To further investigate the role of SOX12 in metastasis, we performed immunohistochemical staining along with a retrospective analysis of clinical cases (Supplementary Fig. S1I). A retrospective review of clinical cases involving SOX12 was also performed which were coordinated with scRNA analysis results (Supplementary Tables S1–3). Therefore, SOX12 is abnormally highly expressed in TC and is associated with high aggressiveness due to its potential activation of EMT via the TGF-β signaling pathway.

### SOX12 enhances the metastasis of PTC cells both in vitro and in vivo

**Fig. 2:**
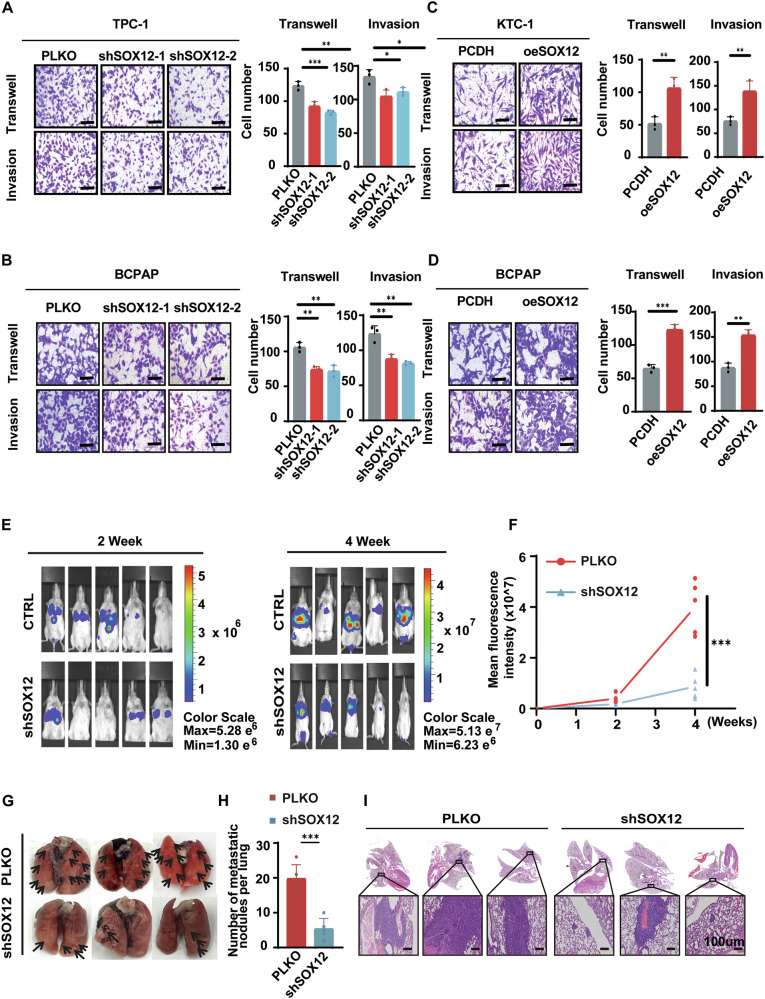
SOX12 enhances the metastasis of PTC cells both in vitro and in vivo. Migration/Invasion assays were used to analyze the effects of SOX12 knockdown (**A**, **B**) or overexpression (**C**, **D**) on the invasiveness of thyroid cancer cell. **E** Representative in vivo bioluminescence images of BCPAP cells transfected with the SOX12 knockdown plasmid. **F** In order to investigate the importance of SOX12 expression in the development and metastasis of PTC tumors, we established a lung metastasis xenograft NCG mouse model by injecting SOX12 knockdown and knockdown control BCPAP cells into the tail vein, respectively. The fluorescence intensity of the SOX12 knockdown group was significantly lower than that of the control group (*N* = 5). **G**–**I** Representative images of lung metastatic foci (**G**). The number of lung metastatic foci was significantly lower in SOX12 knockdown group than in control group (**H**). Metastatic lung was displayed as indicated (**I**). *P* values were determined using a two-tailed unpaired Student’s *t* test (*p < 0.05, **p < 0.01, ***p < 0.001).

To investigate the role of SOX12 in thyroid cancer, we examined SOX12 levels in thyroid cancer cells and found that SOX12 was expressed at much higher levels in thyroid cancer cell lines than in normal human thyroid follicular epithelial cells (Nthy-ori 3-1) (Supplementary Fig. S2A). We subsequently applied lentivirus-mediated *SOX12* knockdown and overexpression in PTC cell lines to clarify the role of SOX12 in the metastasis of PTC (Supplementary Fig. S2B–G). Migration and invasion assays indicated that SOX12 inhibition suppressed the capability of metastasis in PTC cells (Fig. 2A, B). In contrast, SOX12 overexpression increased the migration and invasion of PTC cells (Fig. 2C, D). Consistent with the above findings, wound healing assays also demonstrated that SOX12 remarkably promoted PTC cell migration in vitro (Supplementary Fig. S2H–K). These findings suggest that SOX12 promotes PTC cell migration and invasion in vitro by acting as an oncogene.

To investigate the importance of SOX12 expression in the tumorigenesis and metastasis of PTC, a lung metastasis xenograft nude mouse model was established by injecting *SOX12*-knockdown and *SOX12*-knockdown control BCPAP cells through the tail vein. The results revealed that *SOX12* knockdown reduced PTC lung metastasis at 2 and 4 weeks after cell injection, as measured based on the bioluminescence signals (Fig. 2E, F). On the lung surface, a large number of metastatic lung lesions were observed in the mice receiving knockdown control cells, whereas this number was greatly reduced in the mice implanted with SOX12-inhibited BCPAP cells (Fig. 2G, H). Specifically, the observations were confirmed via haematoxylin and eosin (H&E) staining experiments, which revealed a decreased number of lung metastatic nodules upon SOX12 inhibition (Fig. 2I). These findings indicate that SOX12 functions as an oncogene that promotes PTC cell metastasis in vivo.

### SOX12 transcriptionally activates LDHA expression in PTC

**Fig. 3:**
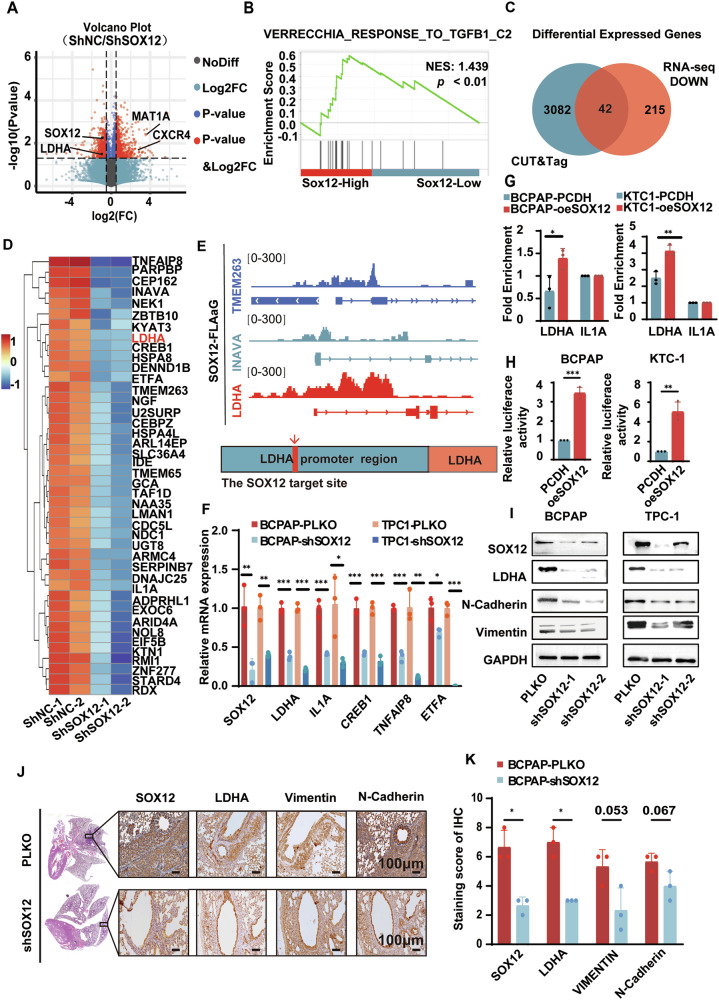
SOX12 transcriptionally activates LDHA expression in PTC. **A** Volcano plot of the RNA-seq analysis of SOX12-knockdown BCPAP cells. **B** GSEA showed that the differentially expressed genes between SOX12-overexpressing cells and control cells were significantly enriched in TGF-beta signaling. Venn diagram (**C**) and heatmap (**D**) demonstrating that 42 genes overlapped among genes that were down-regulated after SOX12 knockdown, as detected by RNA-seq and CUT & Tag. **E** SOX12 occupancy in the vicinity of the LDHA, INAVA, and TMEM263 promoter was assessed by CUT & Tag in SOX12 3×Flag-overexpressing BCPAP cells. **F** RT-qPCR was performed to measure the mRNA expression of 5 representative genes regulated by SOX12. **G** ChIP-qPCR was proformed revealing that SOX12 directly binding to the promoter of LDHA on chr11 18395944 region. **H** luciferase reporter assays showed that SOX12 transactivated LDHA promoter activity. **I** Western blot analysis of LDHA and EMT-related markers, including N-cadherin, Vimentin, in SOX12 knockdown cells. **J**, **K** Representative immunohistochemical images of SOX12, LDHA, N-cadherin and Vimentin staining in metastatic lung tissues (Scale bar is 100 μm). *P* values were determined using a two-tailed unpaired Student’s *t* test. (*p < 0.05, **p < 0.01, ***p < 0.001).

To understand the mechanism of the oncogenic effect of SOX12 in PTC, RNA sequencing was used to identify putative downstream genes regulated by SOX12 in BCPAP cells. Volcano plot analysis revealed that 3672 genes were downregulated, whereas 2452 genes were upregulated after SOX12 inhibition (Fig. 3A). Similarly, GSEA confirmed that SOX12 is positively related to the TGF-β signaling pathway (Fig. 3B). Due to the fact that SOX12 is an important transcription factor that mainly activates the expression of its target genes, CUT&Tag experiments were performed to determine the genome-wide target sites of SOX12 in BCPAP cells to explore the mechanism by which SOX12 regulates gene expression in PTC. A total of 1467 annotated genome-binding peaks were identified, and many peaks (~45.26%) were located in the promoter region of genes located at 0–3 kb around the transcriptional start site, with such peaks exhibiting a high density around the TSS (Supplementary Fig. S3A, B). To further correlate chromatin binding with direct gene regulation, we integrated transcriptome sequencing with CUT&Tag data and noticed that 42 genes, including LDHA, TMEM263 and INAVA, were directly regulated by SOX12 (Fig. 3C–E). In addition, we selected 6 representative genes that are directly regulated by SOX12 and confirmed their expression via qPCR upon *SOX12* knockdown or overexpression (Fig. 3F and Supplementary Fig. S3C). Among these genes, we selected lactate dehydrogenase A (LDHA) as a candidate target gene because our previous study reported that LDHA catalyzed the induction of the EMT process by increasing H3K27 acetylation in PTC [17].

We further investigated whether SOX12 regulated the migration and invasion of thyroid cancer cells by regulating LDHA expression. Chromatin immunoprecipitation (ChIP) qPCR revealed that SOX12 directly binds to the promoter of *LDHA* in the chr11 18395944 region (Fig. 3G). Moreover, luciferase reporter assays revealed that *SOX12* transactivated *LDHA* promoter activity (Fig. 3H). Additionally, Western blotting was used to determine how SOX12 affects EMT and LDHA. The results showed that the protein levels of LDHA, N-cadherin and vimentin were obviously downregulated upon *SOX12* knockdown in PTC cells (Fig. 3I). In contrast, SOX12 overexpression upregulated LDHA, N-cadherin and vimentin expression (Supplementary Fig. S3D). To validate the association between SOX12 and TGF-β secretion, we conducted an ELISA experiment, which demonstrated a positive correlation between SOX12 expression and TGF-β secretion (Supplementary Fig. S3E, F). Importantly, we also performed IHC staining of lung tissue metastasis from the above mentioned mouse model, and the results suggested that SOX12 inhibition reduced both LDHA expression and EMT markers expression in vivo (Fig. 3J, K). Taken together, these results suggest that SOX12 promotes PTC metastasis by directly transactivating LDHA.

### LDHA is required for SOX12-dependent PTC cell metastasis

**Fig. 4:**
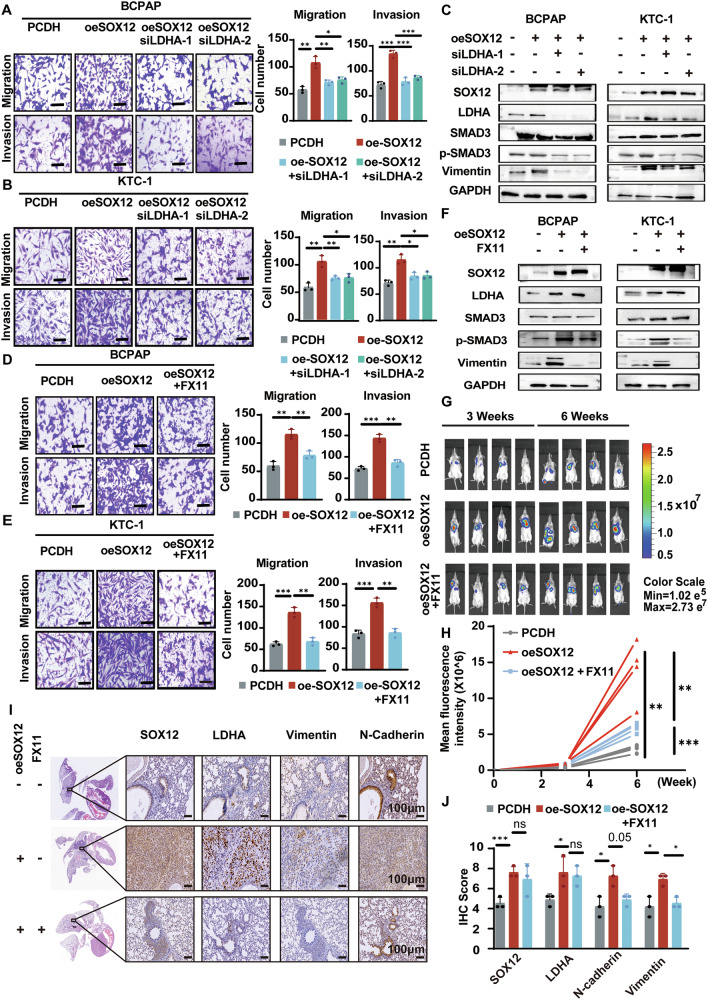
LDHA is required for SOX12-dependent PTC cell metastasis. **A**, **B** Migration/Invasion assays were used to analyze the effects of LDHA interfere in SOX12 overexpressing BCPAP and KTC-1 cells. **C** Western blotting showed LDHA knockdown partially reversed the TGF-β signaling pathway in SOX12 overexpressed PTC cells. **D**, **E** Migration/Invasion assays were used to analyze the effects of treatment with FX11 for 24 h in SOX12 overexpressing BCPAP and KTC-1 cells. **F** The effect of treatment with FX11 for 24 h on the expression of LDHA and EMT markers in SOX12 overexpressing cells was investigated by Western blotting. **G** To verify whether SOX12 exerts its biological function in vivo by regulating LDHA, we injected SOX12-overexpressed BCPAP cells and control cells through the tail vein of NCG mice. After three weeks, the mice were treated with FX11 and DMSO. Representative in vivo bioluminescence images were detected before (3weeks) and after (6weeks) FX11treatment (*N* = 4). **H** The fluorescence intensity of the SOX12 knockdown group was significantly lower than that of the control group. **I**–**J** Immunohistochemical staining of lung metastasis tumors showed that the protein expression levels of vimentin and N-cadherin were reduced in tumors treated with FX11 (**I**–**J**). *P* values were determined using a two-tailed unpaired Student’s *t* test. (*p < 0.05, **p < 0.01, ***p < 0.001).

The transcriptional regulation of LDHA by SOX12 encouraged us to determine whether LDHA was a functional downstream target of SOX12 during its promotion of thyroid cancer metastasis. We subsequently decreased LDHA via siRNA in SOX12-overexpressing BCPAP and KTC-1 cells and found that the loss of LDHA expression significantly attenuated SOX12-dependent cell invasion, migration and wound healing ability (Fig. 4A, B & Supplementary Fig. S4A, B). Western blotting also confirmed that *LDHA* knockdown partially reversed the effects on the TGF-β signaling pathway in SOX12-overexpressing PTC cells (Fig. 4C). Subsequently, we applied the LDHA inhibitor FX11 to SOX12-overexpressing PTC cells, which significantly inhibited LDHA activity [17, 19] Consistent with *LDHA* knockdown, treatment with FX11 also decreased the metastasis and wound healing ability of SOX12-overexpressing BCPAP and KTC-1 cells (Fig. 4D, E & Supplementary Fig. S4C, D). In addition, FX11 treatment attenuated SOX12-induced activation of the TGF-β signaling pathway in PTC cells (Fig. 4F).

To verify whether SOX12 exerts biological functions in vivo by regulating LDHA, we injected SOX12-overexpressing BCPAP cells and control cells through the tail vein in nude mice. Three weeks later, the mice were treated with FX11 and DMSO. The results showed that LDHA inhibition reduced the bioluminescence signals and the tumor sizes of the mice in the BCPAP-SOX12 group (Fig. 4G, H & Supplementary Fig. S4E, F). H&E staining experiments suggested that the numbers of lung metastatic nodules, as well as the visually detected metastatic nodules, were obviously decreased upon FX11 treatment (Fig. 4H & Supplementary S4G). Moreover, immunohistochemical staining of lung metastasis tumors revealed that the protein expression levels of vimentin and N-cadherin were reduced in tumors treated with FX11 (Fig. 4I, J). These observations indicate that LDHA is essential for SOX12-mediated PTC cell metastasis.

### SOX12 and YBX1 cooperate to promote *LDHA* transcriptional activation

**Fig. 5:**
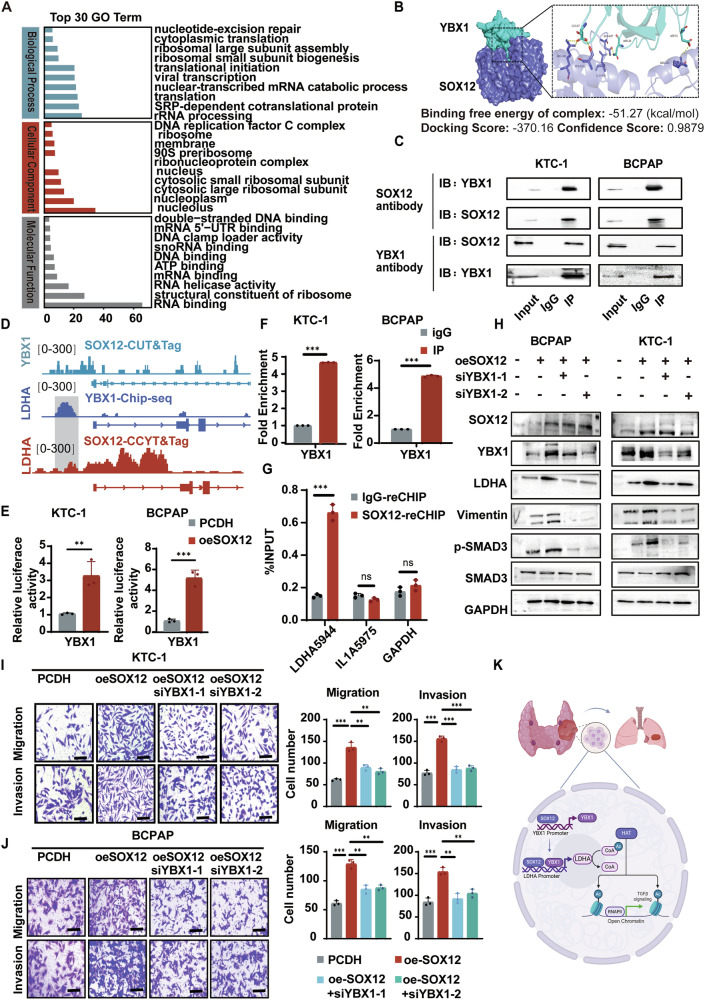
SOX12 and YBX1 cooperate to promote LDHA transcriptional activation. **A** GO analysis results showed that SOX12 involved in transcription and DNA binding process assessed by IP-MS in SOX12 3×Flag-overexpressing BCPAP cells. **B** Protein-protein docking analysis indicating that the binding potential for SOX12 and YBX1. **C** Co-IP assay with SOX12 or YBX1 antibody (or IgG) in BCPAP and KTC-1 cells, followed by immunoblotting of SOX12 and YBX1. **D** SOX12 occupancy in the vicinity of the LDHA and YBX1 promoter was assessed by CUT & Tag in SOX12 3×Flag-overexpressing BCPAP cells, YBX1 occupancy in the vicinity of the LDHA promoter was detected using public data (CistromeDB-62977). **E** Luciferase reporter assays showed that SOX12 transactivated YBX1 promoter activity. **F** ChIP-qPCR was proformed revealing that SOX12 directly binding to the promoter of YBX1. **G** ChIP/re-ChIP assay indicated that YBX1 and SOX12 were bound together on the same LDHA promoter. **H** Western blot analysis of YBX1, LDHA and EMT-related markers, including N-cadherin, Vimentin, SAMD3, P-SMAD3 in SOX12 overexpression cells with or without YBX1 interfere. **I**, **J** Migration/Invasion assays were used to analyze the effects of YBX1 interfere in SOX12 overexpressing BCPAP and KTC-1 cells. **K** Schematic diagram: SOX12 binds the YBX1 promoter to promote YBX1 transcription and interacts with YBX1 to the LDHA promoter to enhance the transcriptional activation of LDHA by SOX12. LDHA subsequently enhances the level of DNA acetylation in thyroid cancer cells, thereby upregulating the TGFbeta signaling pathway and enhancing epithelial mesenchymal transformation of thyroid cancer and promoting metastasis. *P* values were determined using a two-tailed unpaired Student’s *t* test. (*p < 0.05, **p < 0.01, ***p < 0.001).

To better understand the molecular mechanisms by which SOX12 activates LDHA expression, we investigated proteins that interact with SOX12 via the pull-down of a SOX12-Flag fusion protein followed by mass spectrometry analysis. The gene ontology (GO) analysis results revealed that SOX12 is involved in transcription and DNA binding processes (Fig. 5A). Although more than 1000 SOX12-binding proteins have been detected in BCPAP cells, among the transcriptional regulatory proteins, the transcription factor Y-box-binding protein 1 (YBX1) has drawn our attention. As the most prominent member of the YBX family, YBX1 is associated with multiple cancer-related processes [20, 21]. Moreover, protein-protein docking analysis demonstrated the binding potential of SOX12 and YBX1 (Fig. 5B). This analysis indicated that YBX1 interacts with SOX12. To determine whether YBX1 is a protein partner that interacts with SOX12, we immunoprecipitated the SOX12 protein with anti-FLAG antibody in SOX12-overexpressing BCPAP and TPC-1 cells and immunoblotted with anti-YBX1 antibody (Fig. 5C).

To test the possibility that *SOX12* and *YBX1* were assembled on the *LDHA* promoter, we performed ChIP/re-ChIP assays. Intriguingly, the YBX1 re-ChIP assay with anti-SOX12 immunoprecipitates led to the precipitation of the *LDHA* promoter DNA fragment, thus indicating that *YBX1* and *SOX12* were bound together on the same *LDHA* promoter (Fig. 5D, E). These results demonstrate the coexistence of *YBX1* and *SOX12* on the *LDHA* promoter, which is essential for promoting *LDHA* transcriptional activation in thyroid cancer cells.

Next, we analyzed our CUT&Tag data and found that *SOX12* could bind to the promoter region of *YBX1*, thus indicating that SOX12 also activated YBX1 expression (Fig. 5F). ChIP-qPCR analysis found that a higher amount of *SOX12* was accumulated in the *YBX1* promoter of BCPAP and KTC-1 cells when SOX12 was overexpressed (Fig. 5G). To further investigate the role of YBX1 in the regulation of LDHA by SOX12, we conducted rescue experiments. As determined by RT-qPCR and Western blot analysis, YBX1 knockdown blocked the activation of the LDHA/TGF-β pathway induced by SOX12 overexpression in PTC cells (Fig. 5 & Supplementary Fig. S5A, B). These findings suggest that SOX12 positively regulates the expression of LDHA in a YBX1-dependent manner in PTC. Functional experiments showed a reduction in YBX1-antagonized SOX12-regulated metastasis and wound healing ability in PTC cells (Fig. 5I, J & Supplementary Fig. S5C, D). These results indicate that YBX1 plays an essential role in SOX12-induced invasion and EMT in thyroid cancer cells.

### The expression of SOX12 is correlated with YBX1 and LDHA in PTC patients

**Fig. 6:**
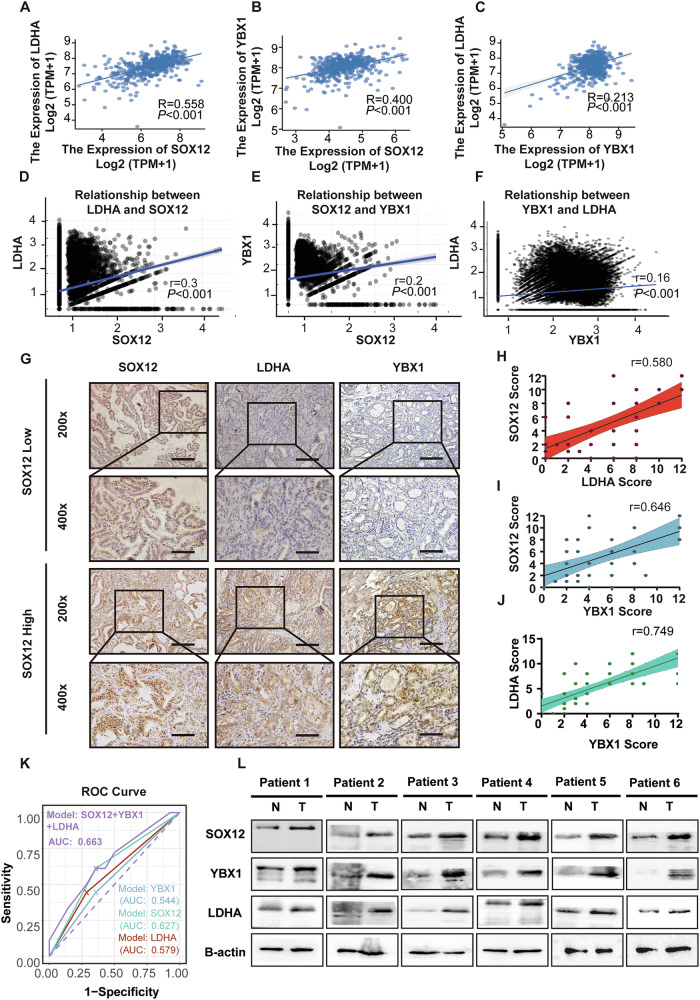
Expression of SOX12 correlates with YBX1 and LDHA in PTC patients. The expression of SOX12 and LDHA or YBX1 scores show a positive correlation within the scRNA-seq (**A**–**C**) and TCGA database (**D**–**F**). **G** Immunohistochemical analysis to evaluate the expression levels of SOX12, YBX1 and LDHA in thyroid cancer tissues with different expressions of SOX12. **H**–**J** The correlation analysis revealed that the higher expression level of YBX1 and LDHA were correlated with higher expression levels of SOX12. **K** A logistic regression model demonstrated the efficacy of SOX12, LDHA, YBX1 expression and combined score in predicting PTC lymph node metastasis. **L** The relative protein expression levels of SOX12, YBX1,LDHA were assessed by western blotting in 6 paired normal thyroid tissues and PTC tissues. *P* values were determined using a two-tailed unpaired Student’s *t* test. (*p < 0.05, **p < 0.01, ***p < 0.001).

To better elucidate the relationships among SOX12, LDHA and YBX1 in PTC, we performed bioinformatic analysis using published data. The expression of SOX12 and LDHA or YBX1 scores were positively correlated according to the scRNA-seq data and TCGA database (Fig. 6A–F). We then used immunohistochemical analysis to evaluate the expression levels of SOX12, YBX1 and LDHA in thyroid cancer tissues with different expression levels of SOX12 (Fig. 6G). Correlation analysis revealed that higher expression levels of YBX1 and LDHA were correlated with higher expression levels of SOX12 (Fig. 6H–J). In addition, we used logistic regression to establish prediction models of SOX12, YBX1, and LDHA expression scores for lymph node metastasis. Compared with these single-factor models, the model combining the three scores had the best prediction efficiency for lymph node metastasis in PTC patients (Fig. 6K). We subsequently compared the protein levels between six pairs of thyroid tumor tissues and matched normal tissues. The results revealed significantly higher SOX12 expression in tumor tissues than in normal tissues, and the expression levels of YBX1 and LDHA were consistent with those of SOX12 (Fig. 6L). In conclusion, the expression level of SOX12 is correlated with YBX1 and LDHA in PTC patients.

## Discussion

The incidence rate of papillary thyroid carcinoma (PTC) is increasing rapidly every year. Although PTC is considered a malignant tumor with a good prognosis, distant metastases occur in a small number of patients, with PTC being the most common cause of death associated with thyroid cancer [22]. Therefore, the understanding of the molecular mechanisms underlying PTC invasion and metastasis is urgently needed. Our results highlighted that SOX12 plays an important role in PTC metastasis and that SOX12 cooperates with YBX1 and promotes oncogenic LDHA expression, thereby facilitating PTC cell migration in vitro and lung metastasis in vivo (Fig. 5K).

SOX12 was identified as being a member of the SOX gene family, which encodes transcription factors. Previous studies have shown that SOX12 plays a crucial role in embryonic development and maintenance of cellular properties [23]. Accumulating evidence suggests that mutations, deletions or overexpression of the SOX12 gene are closely related to the initiation and development of various types of malignant tumors [5]. It has been reported that SOX12 helps in maintaining the tumor characteristics of HCC and induces tumor metastasis through the EMT process in HCC [24].

Furthermore, the long non-coding RNA NR2F1-AS1, which regulates miR-423-5p/SOX12 expression, promoted PTC invasion and metastasis [25]. Although SOX12 is a potential biomarker in PTC, the relationship between SOX12 and metastasis in PTC is still largely unknown. Compared with previous studies, this study demonstrated the prognostic and diagnostic value of SOX12 in PTC via differential expression analysis and survival analysis. Compared with that in normal tissues, SOX12 expression is significantly upregulated in PTC tissues, and its high expression is closely related to poor patient survival. These findings suggest that SOX12 could be a critical biomarker for PTC progression. Despite the compelling findings presented in this study, we acknowledge certain limitations that should be addressed in future work. Notably, the number of independent clinical samples analyzed, particularly those with confirmed pulmonary metastasis, was relatively limited due to the rarity and availability of such specimens.

LDHA is repeatedly associated with altered glycolytic metabolism and is considered to be a potential target for cancer treatment [26]. LDHA has been reported to promote metastasis by facilitating EMT in renal cell carcinoma (RCC), thus suggesting that LDHA may be a promising target for RCC therapy [27]. There is considerable evidence that LDHA combines with other transcription factors and plays an important role in cancer progression. In patients with invasive breast cancer, ErbB2 is positively correlated with the HSF1/LDHA axis [28]. Klf4 binds to the promoter region of *LDHA*, thus regulating LDHA expression in pancreatic cancer cells [29]. Moreover, the downregulation of LDHA promotes cell death via the p53 signaling pathway in breast cancer [30, 31]. The STAT3/LINC00671/LDHA axis regulates glycolysis and proliferation in PTC [32]. Furthermore, the lncRNA GLTC targets LDHA and promotes PTC progression [33]. We previously demonstrated that *LDHA* induces the transcription of EMT-related genes and promotes PTC progression [17]. The data presented in this study provide the first evidence that the transcriptional activation of LDHA is synergistically regulated by SOX12 and YBX1 through the combination of an antagonist at the *LDHA* promoter in PTC. This discovery highlights the critical role of LDHA in PTC metastasis and suggests that the targeting of the SOX12-YBX1-LDHA axis could be a promising therapeutic strategy.

YBX1 is an oncogene which is consistently expressed in various types of cancers [34–36]. In particular, the nuclear localization of YBX1 is associated with a poor prognosis [37, 38]. Jung et al. reported that YBX1 regulates SOX2, thus resulting in a relatively more aggressive subtype of breast cancer [39]. In addition, YBX1 controls p53 to regulate its apoptotic function [40]. However, in PTC, the significance of YBX1 has never been reported. We found that *YBX1* KD antagonized SOX12-regulated metastasis. Furthermore, SOX12 positively regulates the expression of LDHA in a YBX1-dependent manner. Taken together, these findings underscore the importance of YBX1 in PTC progression and suggest that it is a potential therapeutic target that warrants further investigations.

In this study, we demonstrate that pharmacological inhibition of LDHA using FX11 effectively impairs tumor growth and metastatic progression in PTC models, supporting LDHA as a potential therapeutic target within the SOX12-YBX1-LDHA signaling axis. FX11, a small-molecule LDHA inhibitor, has shown promising anti-tumor efficacy in multiple preclinical models [19, 41]. Previous studies have reported that FX11 exhibits favorable pharmacokinetic properties, with reasonable bioavailability and metabolic stability in vivo [19]. To further enhance the translational value of LDHA inhibition, future studies may explore the potential of combination therapies involving FX11. For instance, LDHA inhibition has been reported to synergize with inhibitors targeting complementary metabolic pathways, such as glutaminase or mitochondrial oxidative phosphorylation [42, 43]. Additionally, combining FX11 with conventional chemotherapeutic agents may enhance cytotoxic efficacy by exacerbating metabolic stress in tumor cells. Intriguingly, recent evidence suggests that LDHA inhibition may also modulate the tumor microenvironment by promoting anti-tumor immune responses, providing a rationale for combination with immune checkpoint blockade [44]. These strategies merit further investigation and may offer promising avenues to potentiate the therapeutic impact of LDHA-targeted interventions in PTC and other malignancies.

Metastasis remains the leading cause of cancer-related death, presenting significant challenges despite advances in early detection and treatment. Our findings offer novel insight into the molecular mechanisms underlying PTC metastasis by elucidating the role of the SOX12-YBX1-LDHA signaling axis. The targeting of this signaling node may provide a new avenue for therapeutic interventions in PTC. Future research should focus on further elucidating the precise mechanisms by which SOX12 and YBX1 regulate LDHA and exploring the potential of the targeting of this axis in other cancer types. Additionally, the development of specific inhibitors that disrupt the SOX12-YBX1 interaction or LDHA expression could provide an avenue for new treatment strategies aimed at mitigating PTC metastasis and improving patient outcomes.

## Methods

### Cell lines and cell culture

The TPC-1, KTC-1, BCPAP, and ACT-1 cell lines were purchased from the American Type Culture Collection. The K1, CAL-62, and C643 cell lines were purchased from Guangzhou Cellcook Biotech (China). The Nthy-ori 3-1 cell line was purchased from the Chinese Academy of Science (China). All cell lines have been authenticated by STR analysis. All the cell lines were cultured in RPMI-1640 medium or DMEM medium supplemented with 10% fetal bovine serum (FBS) and maintained in a humidified incubator at 37 °C with 5% CO_2_.

### Clinical data and tissue samples

This study involved tumor tissue sections from 40 patients with complete medical records, diagnosed with PTC, poorly differentiated thyroid carcinoma (PDTC), or ATC at the Tianjin Medical University Cancer Institute and Hospital. Matched fresh PTC tissues and adjacent normal thyroid tissues were obtained from 26 patients. Total RNA was isolated from 17 paired samples, while total protein was isolated from 6 paired samples. This study was approved by the Ethics Committee of Tianjin Medical University Cancer Hospital (Approval ID: Ek2021149), and informed consent was obtained for the use of human tissues in the experiments.

### Total RNA extraction, reverse transcription-quantitative PCR(RT-qPCR)

Total RNA was extracted from thyroid cancer cells and fresh tissues using an RNA extraction solution (G3013; Servicebio, China). cDNA was synthesized from mRNA using the HiScript II 1st Strand cDNA Synthesis Kit (R212-01, Vazyme, China). Quantitative real-time PCR was performed with HiScript II Q RT SuperMix for qPCR (R223-01, Vazyme, China) following the manufacturer’s instructions. Relative RNA expression was calculated using the 2^−ΔΔCt^ method, with β-actin serving as the internal control. Primer sequences are listed in Additional file 1.1.

### Western blotting

Cells or tissues were lysed with RIPA buffer (R0020, Solarbio, China), and protein concentrations were determined using the BCA Protein Assay Kit (PC0020, Solarbio, China). The samples were prepared for SDS-PAGE by mixing with protein sample loading buffer (LT101, Epizyme, China) and boiling. Proteins were transferred to PVDF membranes (WJ002, Epizyme, China) and blocked with 5% skim milk (D8304, Solarbio, China) in TBS-T for 1 h. The membranes were incubated with primary antibodies at 4 °C overnight, followed by incubation with HRP-conjugated secondary antibodies for 1 h. Chemiluminescence detection was performed using the Omni-ECL Femto Light Chemiluminescence Kit (SQ201, Solarbio, China).

### Cell transfection and lentivirus infection

Small interfering RNAs (siRNAs) or plasmids were transfected into cells using Lipofectamine 3000 (L3000075, Thermo Fisher Scientific, USA), and siRNAs were synthesized by Hanbio Biotechnology (China). Lentiviruses for knockdown and overexpression (shSOX12, OE-SOX12) were purchased from Hanbio Biotechnology (China). The lentivirus-infected cancer cells were selected with 1 μg /mL puromycin. The sequences of the siRNAs and shRNAs that were used are listed in Supplementary Additional File 1.2.

### Mice models and treatments

For the in vivo thyroid cancer lung metastasis model, 4–5 weeks NCG mice were purchased from SPF Biotechnology (Beijing, China) and bred in specific pathogen-free (SPF) conditions. 1 × 10^6^ BCPAP cells in 100 μL PBS were intravenously (i.v.) inoculated to the tail vein of mice. Nine days later, the mice were treated with 2% DMSO, FX11 (3 mg/kg) for 3 weeks. For the in vivo imaging, we used BCPAP-luci cells stably expressing luciferase, and luciferase substrate D-Luciferin was retro-orbitally injected before imaging at days 21 and 42. After 6 weeks, mice were sacrificed and the lungs were collected and fixed with Bouin’s solution to count the number of metastatic pulmonary nodules, the lungs were embedded in paraffin for subsequent hematoxylin and eosin (H&E) staining. All animal experiments were approved by the Ethics Committee of the Tianjin Medical University Cancer Institute and Hospital (Approved No.: NSFC-AE-2021199).

### RNA-sequencing analysis

*SOX12*-knockdown or control BCPAP cells were lysed using an RNA extraction solution (G3013; Servicebio, China) and the mRNA was extracted according to the manufacturer’s protocol. The global gene expression profiles were determined by mRNA sequencing, which was performed at Majorbio Bio-Pharma Technology (China). Bioinformatic analyses were performed as previously described [46]. The raw RNA-Seq data were uploaded to the Sequence Read Archive (SRA) under accession number PRJNA1172414.

### CUT&Tag assay

The CUT&Tag assay was conducted using the Hyperactive Universal CUT&Tag Assay Kit for Illumina (TD903, Vazyme, China). In brief, *SOX12*-knockdown or control BCPAP cells were harvested. Con A magnetic beads were used for cell capture, while 5% Digitonin was utilized to permeabilize the cell membrane. The DNA sequences that bound to the target protein were specifically cleaved by combining primary antibodies, secondary antibodies, and the Protein A-Transposome. Subsequently, PCR was employed to assemble a second-generation sequencing library, enabling the acquisition of a high-resolution map of the genes bound to the target protein via next-generation sequencing. Libraries were sequenced on a NovaSeq PE150 by Novogene (Beijing, China). CUT&Tag data were aligned to the GRCh37/hg19 human reference genome using SOAPaligner/SOAP2 (Short Oligonucleotide Analysis Package). A maximum of two mismatches were allowed in a pair. Reads that mapped only once at a specific location were used for peak calling. The peaks from CUT&Tag were identified using MACS software, specifically version MACS-1.4.2. Big-wig files were generated using MACS-1.4.2. CUT&Tag tracks were visualized using IGVtools (version 2.11.3). The raw CUT&Tag data were uploaded to the Sequence Read Archive (SRA) under accession number SAMN44286703.

### Chromatin immunoprecipitation (ChIP)-qPCR/ChIP-ReChIP-qPCR

For the first ChIP sample, 1 × 10^6^ cells were crosslinked with 1% formaldehyde for 10 min at RT, followed by quenching with 125 mM glycine. Cell pellets were collected and lysed in lysis buffer, followed by sonication to shear chromatin into DNA fragments between 200 and 500 bp. Chromatin fragment were immunoprecipitated with SOX12 antibody (proteintech, 23939-1-AP) or IgG at 4 °C overnight, followed by incubated with protein A magnetic beads. The protein/DNA complexes were then separated and purified DNA was used for qPCR analysis. For the re-ChIP sample, based on the first ChIP elution sample, YBX1 antibody (proteintech, 20339-1-AP) was added, and the immunoprecipitation experiment was repeated to study the synergistic regulation of downstream target genes by two or more transcription regulatory proteins. The sequences of the primers that were used for ChIP-qPCR are listed in Supplementary Additional File 1.3.

### Luciferase reporter assay of SOX12 binding sites

Wild-type and mutant SOX12 consensus sequences were synthesized by (Hanbio, China) and fused with a firely luciferase reporter. The adenosine (A) in the SOX12 motif was replaced by thymine (T). BCPAP and KTC-1 cells were seeded into a 24-well plate followed by co-transfection of 0.5 μg of wild-type or mutated SOX12 reporter plasmids and 20 ng renilla luciferase reporter vector. After 24 h, cells were harvested to access the luciferase activity using the Dual-Glo Luciferase system (Promega) with the normalization to pRL-TK. Each group was conducted in triplicate.

### Statistical analysis

The experimental data were analyzed using the GraphPad Prism 10.0 software. All the in vitro results were representative of at least three independent experiments and were presented as the mean ± SD or median with range. Paired PTC and corresponding normal thyroid samples were analyzed using paired Student’s *t* test. Correlations between different genes were analyzed using the Pearson correlation test. Kaplan–Meier analysis was used to evaluate survival curves, and the differences in the survival probabilities were assessed using the log-rank test. Differential analysis between 2 groups was conducted with the two-tailed unpaired Student’s *t* test (***p < 0.001, **p < 0.01, *p < 0.05).

Detailed information about the other experiments including Thyroid Differentiation Score (TDS) calculation, analysis of single-cell datasets, and immunohistochemistry (IHC) are included in Supplementary Additional File 2.

## Supplementary information

Westernblot_rawdata

suppltmentary figures-doc

## Data Availability

The authors confirmed that the data supporting the findings of this study are available within the article. Sequencing reads are available from the Sequence Read Archive under BioProject ID PRJNA1172414. Uncropped Western blot images can be found in Supplementary materials. All additional data are available from corresponding authors upon request, please contact xzheng05@tmu.edu.cn.
