# Supplementary figures and images for: The SOX12-YBX1-LDHA signaling axis drives metastasis in papillary thyroid carcinoma
Source: Cell Death Dis. 2025 Jul 1;16(1):474. doi: 10.1038/s41419-025-07797-5 (PMC12215885; doi:10.1038/s41419-025-07797-5)

Fig3

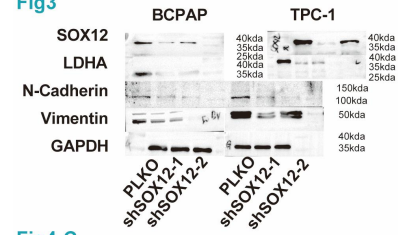

Fig5-C

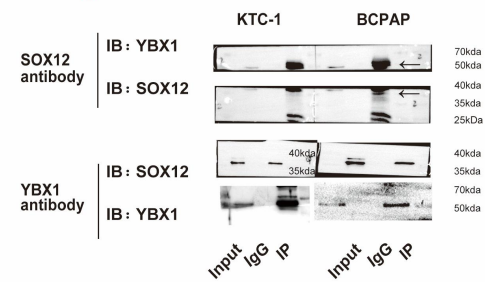

Fig4-C

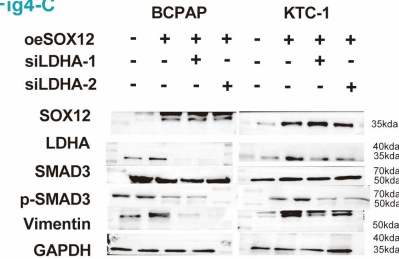

Fig5-H

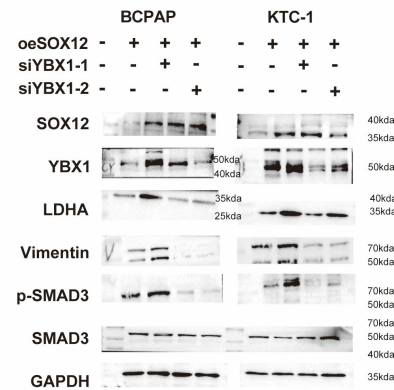

Fig4-F

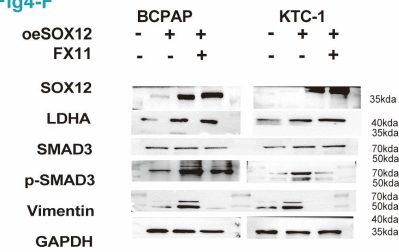

Fig6-L

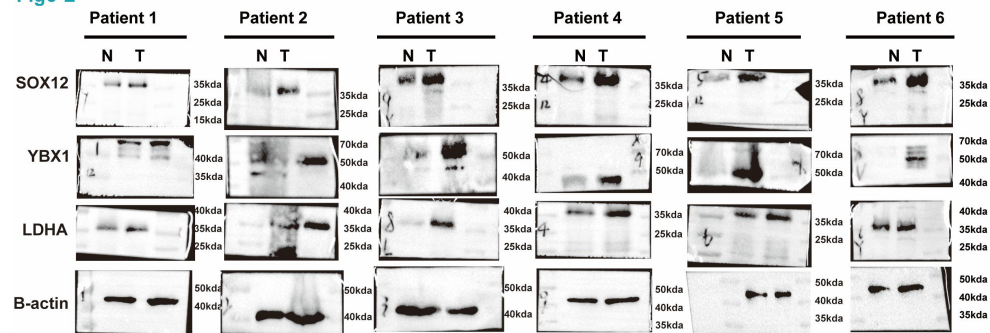

FigS2

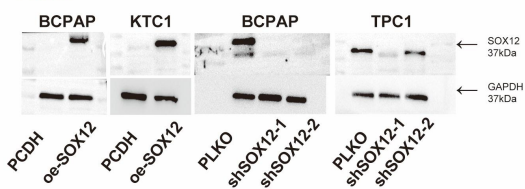

FigS3

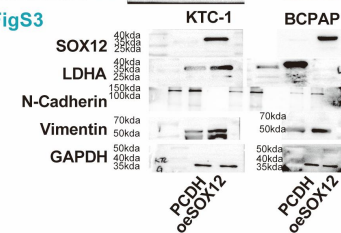

Supplement: Supplementary file 1 — Westernblot_rawdata [file 41419_2025_7797_MOESM1_ESM.pdf]
