# Supplementary material for: The SOX12-YBX1-LDHA signaling axis drives metastasis in papillary thyroid carcinoma
Source: Cell Death Dis. 2025 Jul 1;16(1):474. doi: 10.1038/s41419-025-07797-5 (PMC12215885; doi:10.1038/s41419-025-07797-5)
Supplement: Supplementary file 2 — suppltmentary figures-doc [file 41419_2025_7797_MOESM2_ESM.docx]

Supplementary Materials

**The SOX12-YBX1-LDHA signaling axis drives metastasis in papillary thyroid carcinoma**

Xianhui Ruan^1#^, Yue Huang^1#^, Yu Zeng^1^, Zhenhao Zhao^2^, Mei Tao^1^, Zewei Zhao^1^, Yuqi Wang^1^, Guangwei Xu^1^, Wei Zhang^3,4^, Jialong, Yu^1^, Wei Luo^1^, Songfeng Wei^1^, Xichuan Li^5^, Ming Gao^3,4^, Yang Yu^1*^, Peng Li^2*^, Xiangqian Zheng^1*^

^1^ Department of Thyroid and Neck Tumor, Tianjin Medical University Cancer Institute and Hospital, National Clinical Research Center for Cancer, Key Laboratory of Cancer Prevention and Therapy, Tianjin’s Clinical Research Center for Cancer, Tianjin, 300060, China

^2^ State Key Laboratory of Medicinal Chemical Biology, College of Life Sciences, Nankai University, 300071 Tianjin, China

^3^Department of Thyroid and Breast Surgery, Tianjin Key Laboratory of General Surgery in Construction, Tianjin Union Medical Center, Tianjin 300121, China

^4^ School of Medicine, Nankai University, Tianjin, China

^5^Tianjin Key Laboratory of Animal and Plant Resistance, College of Life Sciences, Tianjin Normal University, Tianjin, China

^#^Xianhui Ruan and Yue Huang contributed equally to this work and share first authorship.

***Corresponding author:** Xiangqian Zheng, xzheng05@tmu.edu.cn;

Peng Li, lipeng@nankai.edu.cn;

Yang Yu, nkyuyang@126.com

Supplementary Figures

**
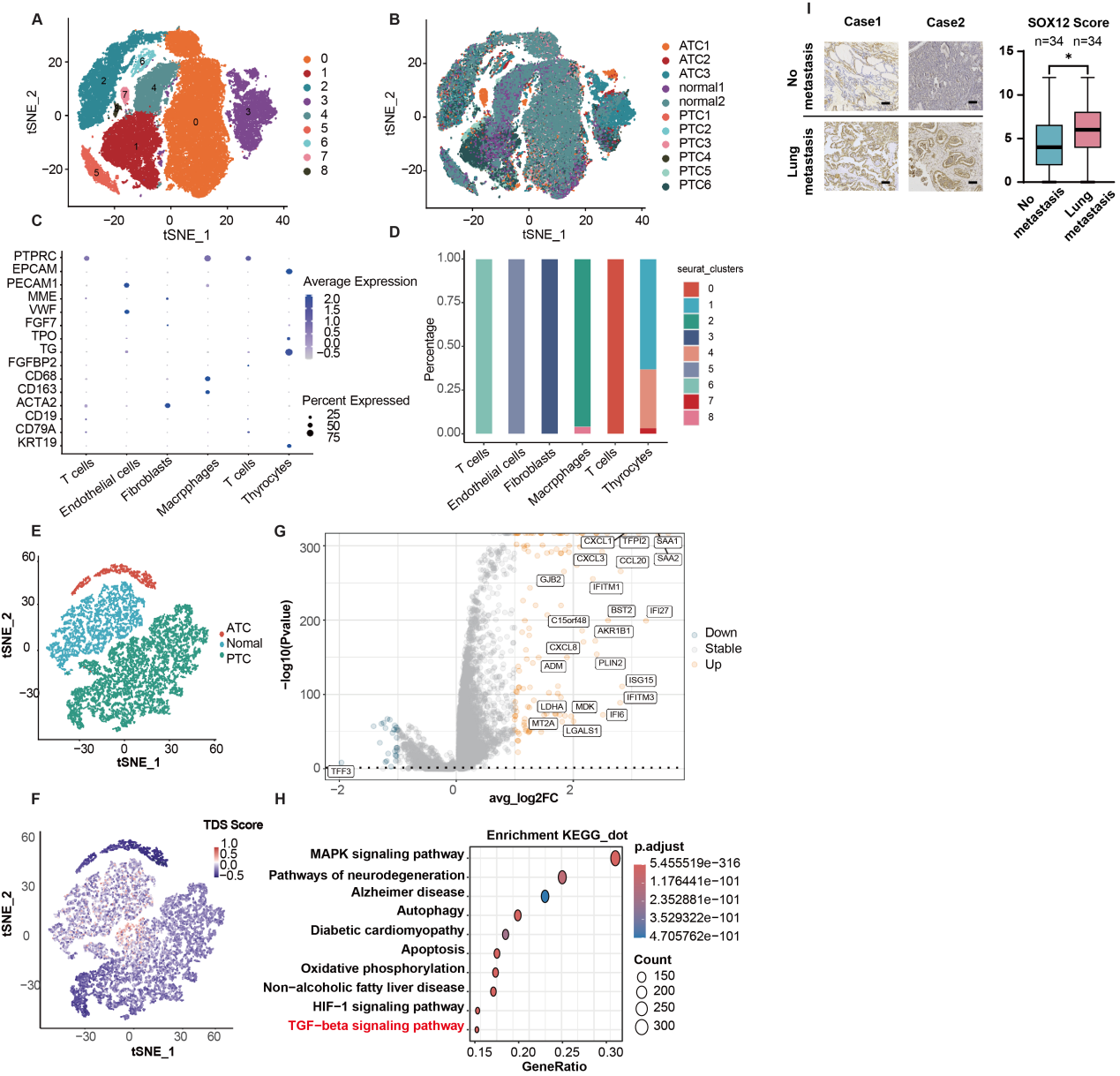
**

**Figure S1. Signal cell RNA-seq analysis identifies SOX12 as a metastasis associated gene for PTC.** (A) TSNE plot of 9 groups of thyroid cells (normal + tumor) obtained by dimensionality reduction clustering. (B) TSNE plot of 11 samples of thyroid cells (normal + tumor) obtained by dimensionality reduction clustering. (C) Bubble plot showing the expression of marker genes in cells from different groups. (D) The proportion of cell clusters in each sample. (E) Dimensionality reduction clustering results for thyroid follicular cells and tumor cells. (F) Verification of the grouping of thyroid follicular cells and tumor cells by examining differences in TDSs. (G) Volcano plot depicting the representation of differential expressed signature genes of the tumor cells based on SOX12 expression. (H) KEGG annotation showing top enriched pathways in tumor cells with SOX12 expression. (I) Correlation between SOX12 score and pulmonary metastasis in PTC: Representative IHC images of 4 primary tumor samples (left), and SOX12 IHC scores in 34 pulmonary metastatic PTC cases and 34 non-metastatic clinical cases (right).


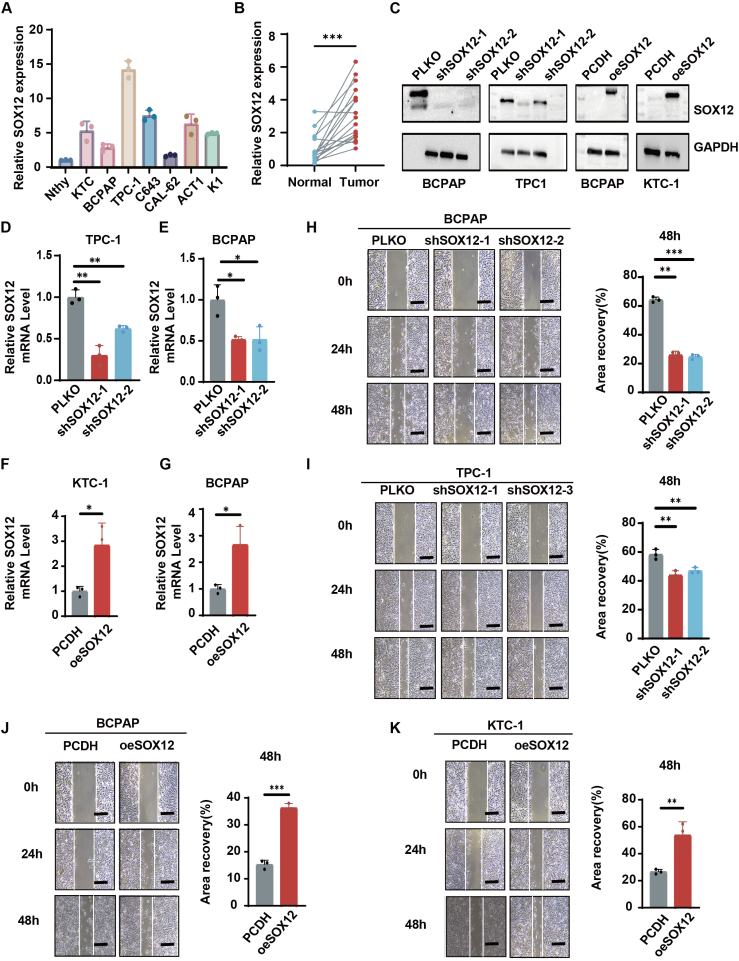


**Figure S2. SOX12 enhances the metastasis of PTC cells both in vitro and in vivo.** (A) RT-qPCR was performed to measure the mRNA expression of SOX12 between thyroid cancer cell lines and normal human thyroid follicular epithelial cells. (B) The relative mRNA expression levels of SOX12 were assessed by qPCR in 17 paired normal thyroid tissues and PTC tissues. (C) Western blotting was used to analyze the effects of SOX12 overexpression or knockdown. (D-G) RT-qPCR was performed to measure the mRNA expression of SOX12 overexpression or knockdown. **(**H-K) Wound-Healing assay were used to analyze the invasiveness of SOX12 overexpression or knockdown. P values were determined using a two-tailed unpaired Student’s t-test. (*p < 0.05, **p < 0.01, ***p < 0.001).


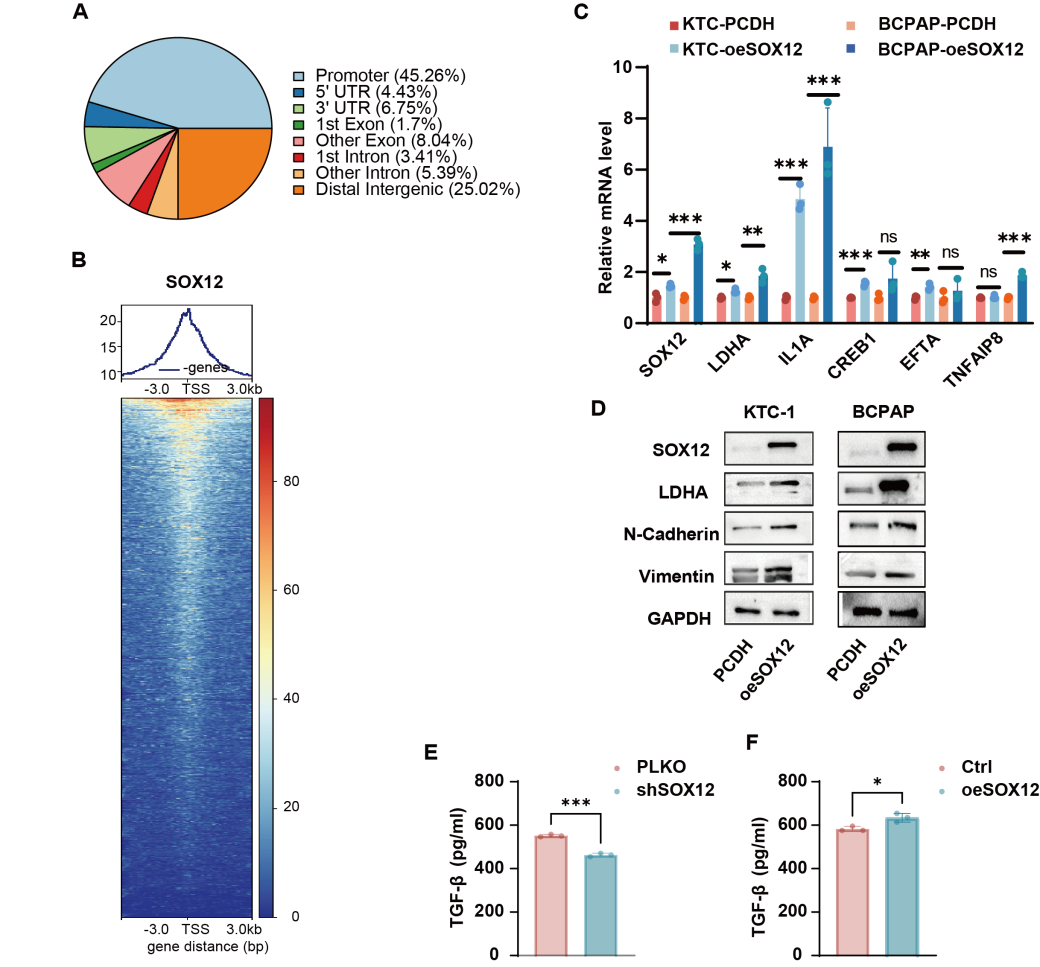


**Figure S3. SOX12 transcriptionally activates LDHA expression in PTC.** (A)Distribution of 1467 SOX12 binding sites in the genome based on SOX12-3×Flag CUT&TAG peak maps. (B) Heatmap of CUT&TAG signal within SOX12-3×Flag peaks in BCPAP cells. (C)RT-qPCR was performed to measure the mRNA expression of 5 representative genes regulated by SOX12 overexpression. (D) Western blot analysis of LDHA and EMT-related markers, including N-cadherin, Vimentin, in SOX12 overexpression cells. (E-F) ELISA analysis of TGF-β in SOX12 knockdown cells and overexpression cells. P values were determined using a two-tailed unpaired Student’s t-test. (*p < 0.05, **p < 0.01, ***p < 0.001)


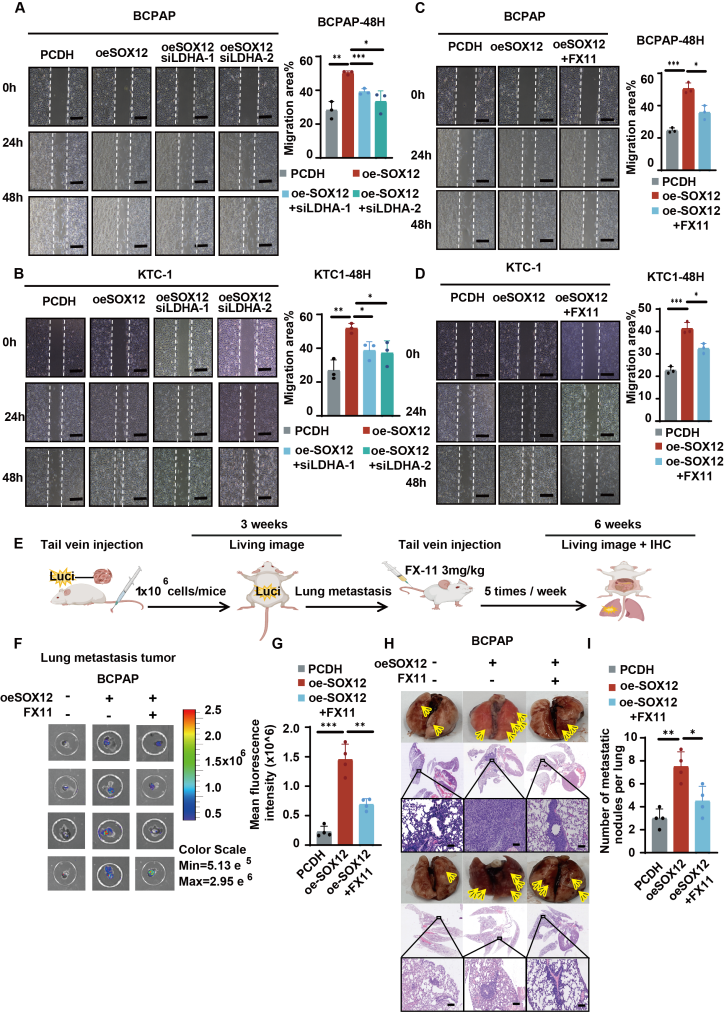


**Figure S4. LDHA is required for SOX12-dependent PTC cell metastasis.** (A-B) Wound-Healing assay were used to analyze the invasiveness of SOX12 overexpression with or without LDHA knockdown. (C-D) Wound-Healing assay were used to analyze the invasiveness of A-SOX12 overexpression with or without FX11 treatment. (E) The diagrammatic sketch of constructing tail-vein injection of BCPAP cells in NCG mice to generate experimental pulmonary metastasis, followed by treatment with the indicated drug. n = 4 mice per group. (F-H) In vivo bioluminescence images of lung specimen after BCPAP cells transfected with the SOX12 overexpressing plasmid with or without FX11 treatment (F). The fluorescence intensity of the SOX12 overexpression group was significantly higher than that of the control group, while FX11 treatment partially inhibit lung metastasis (G). Mice were killed on week 6 after injection and metastases were quantified in excised lungs (arrow marked the metastasis location), metastatic tumors were analyzed by HE staining of excised lungs (H). The number of lung metastatic foci was significantly lower in SOX12 knockdown group than in control group (I). Scale bar= 100 μm. Error bars, mean ± SEM (n = 4 independent experiments), *p < 0.05, **p < 0.01, ***p < 0.001.


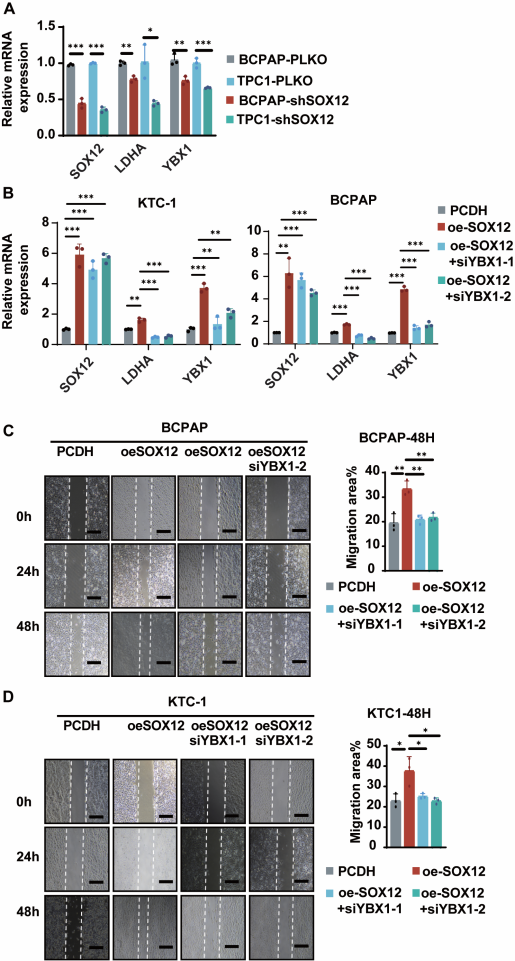


**Figure S5. SOX12 and YBX1 cooperate to promote LDHA transcriptional activation.** (A) RT-qPCR resulted the expression of LDHA and YBX1 are associated with SOX12 expression. (B) RT-qPCR indicated the expression of LDHA was down-regulated after YBX1 knockdown. (C-D) Wound-Healing assay were used to analyze the invasiveness of SOX12 overexpression with or without YBX1 knockdown. Scale bar= 100 μm. P values were determined using a two-tailed unpaired Student’s t-test. (*p < 0.05, **p < 0.01, ***p < 0.001)

**Table S1. Univariate analysis of clinicopathological features and SOX12 expression between Lung metastasis status**

|  |  | | | Lung metastasis status | | | | | | |  | | |  |
| --- | --- | --- | --- | --- | --- | --- | --- | --- | --- | --- | --- | --- | --- | --- |
| Variables | Total | | | No metastasis | | | Lung metastasis | | | | X^2^ | | | *P* |
|  |  | | | ( n = 34) | | | ( n = 34) | | | |  | | |  |
| Age |  |  | | |  | | |  |  | | | |  | |
| <55 | 50 | | | 29 | | | 21 | | | | 4.835 | | | **0.027** |
| >=55 | 18 | | | 5 | | | 13 | | | |  | | |  |
| Sex |  | | |  | |  |  | | |  | |  | |  |
| Female | 41 | | | 21 | | | 20 | | | | 0.061 | | | 0.804 |
| Male | 27 | | | 13 | | | 14 | | | |  | | |  |
| Multifocality | | |  | | | |  | | |  | |  | |  |
| Present | 8 | | | 2 | | | 6 | | | | 2.267 | | | 0.132 |
| Absent | 60 | | | 32 | | | 28 | | | |  | | |  |
| T stage | | |  | | |  |  | | |  | |  | |  |
| T1/2 | 43 | | | 26 | | | 17 | | | | 5.123 | | | **0.023** |
| T3/4 | 25 | | | 8 | | | 17 | | | |  | | |  |
| SOX12 expression | | | |  | | |  | | | |  | | |  |
| SOX12 Low | 27 | | | 18 | | | 9 | | | | 4.976 | | | **0.026** |
| SOX12 High | 41 | | | 16 | | | 25 | | | |  | | |  |

**Table S2. Univariate analysis of clinicopathological features and SOX12 expression**

|  |  | | | SOX12 expression | | | | | | |  | | |  |
| --- | --- | --- | --- | --- | --- | --- | --- | --- | --- | --- | --- | --- | --- | --- |
| Variables | Total | | | Low expression | | | High expression | | | | X^2^ | | | *P* |
|  |  | | | ( n = 74) | | | ( n = 81) | | | |  | | |  |
| Age |  |  | | |  | | |  |  | | | |  | |
| <55 | 118 | | | 54 | | | 64 | | | | 0.776 | | | 0.378 |
| >=55 | 37 | | | 20 | | | 17 | | | |  | | |  |
| Sex |  | | |  | |  |  | | |  | |  | |  |
| Female | 111 | | | 56 | | | 55 | | | | 1.150 | | | 0.284 |
| Male | 44 | | | 18 | | | 26 | | | |  | | |  |
| Multifocality | | |  | | | |  | | |  | |  | |  |
| Present | 11 | | | 8 | | | 3 | | | | 2.963 | | | 0.085 |
| Absent | 144 | | | 66 | | | 78 | | | |  | | |  |
| T stage | | |  | | |  |  | | |  | |  | |  |
| T1/2 | 112 | | | 59 | | | 53 | | | | 3.944 | | | **0.047** |
| T3/4 | 43 | | | 15 | | | 28 | | | |  | | |  |
| N stage | | |  | | | | | | | |  | | |  |
| N0 | 17 | | | 12 | | | 5 | | | | 3.995 | | | **0.046** |
| N1a/b | 138 | | | 62 | | | 76 | | | |  | | |  |
| TNM stage | | |  | | | |  | | |  | |  | |  |
| I+II | 121 | | | 65 | | | 56 | | | | 7.899 | | | **0.005** |
| III+IV | 34 | | | 9 | | | 25 | | | |  | | |  |

**Table S3. Multivariate COX regression analysis of RFS and OS in relation to clinicopathological features**

| Variables | Recurrence-free survival |  | Overall survival |  |
| --- | --- | --- | --- | --- |
|  | Hazard ratio (95% CI) | *P* | Hazard ratio (95% CI) | *P* |
| Age>=55 | 0.484 (0.104-1.648) | 0.289 | 2.442 (0.315-13.868) | 0.330 |
| Female | 0.503 (0.203-1.259) | 0.136 | 0.267 (0.054-1.240) | 0.090 |
| III+IV | 1.538 (1.102-2.164) | **0.012** | 1.777 (1.034-3.180) | **0.040** |
| Multifocality | 3.984 (0.973-17.314) | 0.055 | 3.355 (0.385-23.099) | 0.220 |
| SOX 12 expression | 1.183 (1.039-1.358) | **0.013** | 1.423 (1.130-1.870) | **0.005** |

Additional File 1

1. Primers for RT-qPCR

| Name | Forward Primer (5'-3') | Reverse Primer (5'-3') |
| --- | --- | --- |
| β-actin | GATCATTGCTCCTCCTGAGC | ACTCCTGCTTGCTGATCCAC |
| SOX12 | AAGAGGCCGATGAACGCATT | TAGTCCGGGTAATCCGCCAT |
| LDHA | CGTGTTATTGGAAGCGGTTG | TTCATTCCACTCCATACAGGC |
| IL1A | TGGTAGTAGCAACCAACGGGA | ACTTTGATTGAGGGCGTCATTC |
| CREB1 | ATTCACAGGAGTCAGTGGATAGT | CACCGTTACAGTGGTGATGG |
| TNFAIP8 | ATAGACGACACAAGTAGTGAGGT | CCACGGTCATAGCAAGCTGAT |
| ETFA | TGGCGGTAGTGCCAGTTCAGAA | CTCTCCACTCTTCAAGCCTCGA |
| YBX1 | TGCAGCAGACCGTAACCATT | TGGATCGGCTGCTTTTGTC |

1. Sequences of plasmid, shRNA and siRNA

| Plasmids | Forward Primer (5'-3') | Reverse Primer (5'-3') |
| --- | --- | --- |
| oeSOX12 | TACGACTCACTATAGGCTAGCATGGTGCAGCAGCGGGGCGCGAG | ATCTCCTCCTCCAGCGGCCGCGTAGGTGAAAACCAGGTCTGCGAT |
| shSOX12-1 | CCGGCATGGCGGATTACCCGGACTACTCGAGTAGTCCGGGTAATCCGCCATGTTTTTG | AATTCAAAAACATGGCGGATTACCCGGACTACTCGAGTAGTCCGGGTAATCCGCCATG |
| shSOX12-3 | CCGGCCCGTCTAGCATCGCAGACCTCTCGAGAGGTCTGCGATGCTAGACGGGTTTTTG | AATTCAAAAACCCGTCTAGCATCGCAGACCTCTCGAGAGGTCTGCGATGCTAGACGGG |
| siRNA | Sense Sequence | Anti-Sense Sequence |
| siLDHA-1 | GCUGAUUUAUAAUCUUCUAAA | UAGAAGAUUAUAAAUCAGCUG |
| siLDHA-3 | CAGAUUUAGGGACUGAUAAAG | UUAUCAGUCCCUAAAUCUGGG |
| siYBX1-1 | UUUGCUGGUAAUUGCGUGGAGGACC | GGUCCUCCACGCAAUUACCAGCAAA |
| siYBX1-2 | GGUCAUCGCAACGAAGGUU | AACCUUCGUUGCGAUGACC |

1. Primers for ChIP-qPCR and Luciferase assay

| Name | Forward Primer (5'-3') | Reverse Primer (5'-3') |
| --- | --- | --- |
| LDHA-5299 | GCATGCAAAGATGAAGCAGG | CTAGCTTTCGGTCTCCCTATTTG |
| LDHA-5944 | TCTACACTCCCTGTACTGAAAC | CGCCAGACTTTAAATCGCAATAC |
| LDHA-luc | CTAGCTAGCCAACTTCAGCTCTCTGCCTT | CCCAAGCTTGCGCGTGGCAATGAGAT |
| IL1A-5927 | CCTGTTTTACAACTCTGTCATGC | CATCTCCACCTGCCTTCC |
| IL1A-5975 | CCACACTTGGATGTAAGCAATG | CATTTCTGTGTTGCCTAAAGAGG |
| IL1A-5756 | TTTCTCACGCCCCATTCC | TGCTAGGATAGACTCGTCCAG |
| TNFAIP8-3468 | GCTCTGTAATGGGCTCAGTTAG | AGGTGCAAGTCAAGGTATGTAG |
| TNFAIP8-6996 | CCCCTTCTATCTCCTTTAGATCTTATG | TTCTTCTATACTTAGCACTGGCC |
| TNFAIP8-2989 | TCTCGAAACTTGGAAGGCTG | GTACAGCTATTCACGGGAGAAC |
| ETFA-9284 | CTGGGCTCTCAAAACTGTCTAG | AGACTGGGTAATAGAGGGAGAC |
| ETFA-1635 | CAAAGGTGAGGCTATTCCCC | AGTACAGTCTGAGCTCGGTC |
| ETFA-1374 | CTGAGTACAGGAGCAATGTCTG | ACTTCCCTTATTGGTGTCAGC |
| YBX1-Pro-1 | GGCACATACCTGTAGTCCTAAC | GAGTGTAGTGGCACGATCATAA |
| YBX1-Pro-2 | AGGGTGGGATTGTAAAGAGATG | AGGACAGAGACTGGCATAGA |
| YBX1-Pro-3 | TTCCAAGGATGTTCCCAGAAG | AGGATGCTCTGAGGTAAGTAGA |
| YBX1-Pro-4 | GGCAGTTGTAGCAAACCTAAC | TCTGGGAACATCCTTGGAATAA |
| YBX1-Pro-5 | GCACTTTCTGTTTGCTCTCATT | CTGTTTGCTACCTTTGCTCTTG |
| YBX1-luc | CGACGCGTGTTACAAGCACCTGGAAGGTT | CCGCTCGAGTGACTGGGGCCGGCTGCGGCA |

1. Antibodies and drugs used in this study

| Antibodies name | Application | Supplier | Cat # |
| --- | --- | --- | --- |
| Anti-human SOX12 antibody | WB, IHC | Proteintech | 23939-1-AP |
| Anti-human LDHA antibody | WB, IHC | Proteintech | 21799-1-AP |
| Anti-human YBX1 antibody | WB, IHC | Abways | CY5462 |
| Anti-human SMAD3 antibody | WB | Cell Signaling Technology | A5549 |
| Anti-human PSMAD-3 antibody | WB | Cell Signaling Technology | sc-518211 |
| Anti-human Vimentin antibody | WB, IHC | Cell Signaling Technology | 24324-1-AP |
| Anti-human N-Cadherin antibody | WB, ChIP, CUT&Tag | Cell Signaling Technology | Ab9049 |
| Anti-human β-actin antibody | WB | ABclonal Technology | AC026 |
| Anti-human Ki67 antibody | IHC | Cell Signaling Technology | 9449 |
| Anti FLAG antibody | WB, ChIP, CUT&Tag | Cell Signaling Technology | 14793 |
| Anti-human GAPDH antibody | WB | Santa Cruz Biotechnology | sc-47724 |
| Normal Rabbit IgG | ChIP, CUT&Tag | Cell Signaling Technology | 2729 |
| Puromycin 2HCl | / | Selleck | S7417 |
| FX11 | / | Selleck | S8928 |
| D-Luciferin (potassium salt) | / | APExBIO | C3654 |

Additional File 2

**Analysis of single-cell datasets**

The single-cell RNA-sequencing (scRNA-seq) datasets, GSE148673, GSE184362, and GSE134355, containing ATC, PTC, or healthy thyroid samples, were acquired from the GEO database. Briefly, the Seurat package was used for processing scRNA-seq data, including data filtering (cells and genes), normalization, principal component analysis (PCA), and t-distributed stochastic neighbor embedding(t-SNE). For quality control, we removed single cells that had fewer than 300 genes or more than 8,000 genes for diminishing ruptured cells and potential non-singlet cells, respectively. Given the common problem of low viability when single cells are isolated from tissues, we then filtered out cells with more than 10% mitochondria and more than 3% HB genes to remove dying cells and blood cells. We set the minimum level of UMI count of each cell at 100,000 to illuminate low-depth data. Thus, 66,061 cells and 42,097 genes were included in this analysis. Subsequently, we used Harmony algorithm to integrate and remove batch effect. After cell filtering, the scRNA-seq data of high-quality cells were normalized to find highly variable genes for downstream analyses. Then, PCA was done on highly variable genes to identify significant principal components (PCs). Cell clustering was undertaken on the top-10 PCs using the t-SNE algorithm. Next, annotation of cell type in different cell clusters was done with the SingleR package for automatic cell annotation and obtained 6 major cell subsets. We also used representative marker genes to verify the accuracy of each cell type classification.

**Cell cluster regrouping and Identification of DEGs**

First, thyroid cells were re-grouped according to sample source, and TDS scores were used to verify the accuracy of classification using Seurat’s AddModuleScore function. Subsequently, we divided tumor cells into high-expression and low-expression groups based on whether or not they expressed SOX12. The differential expressed genes (DEGs) in each sub-cluster were identified through the FindMarkers function in Seurat. The significance levels of these signature genes were determined using the Wilcoxon rank-sum test along with Bonferroni correction. GSVA analysis was performed using GSVA packages to enrich the significant pathways depends on SOX12 expression. We also used clusterProfiler packages to enrich the KEGG and GSEA signaling pathway of the above differential genes, and concentrated the related pathways in the high-expression and low-expression SOX12 groups. Cell trajectory analysis and cell communication analysis are also used to elucidate the bio-function of SOX12 gene in tumor evolution and cancer microenvironment, respectively.

**Clustering and survival analysis of TCGA thyroid tumor and normal samples**.

The bulk-RNA-seq data was obtained from The Cancer Genome Atlas database and GTEx database. Non-parametric rank Test (Wilcox Test) and R software (version 4.1.2) Beeswarm package were used to analyze the expression difference of SOX12 mRNA obtained from the TCGA database in thyroid cancer and para-cancer tissues. P≤0.05 was considered statistically significant. R-packet ggpubr was used to analyze the relationship between SOX12 gene and clinicopathological features of thyroid carcinoma. All cancer cases were divided into high and low-expression groups according to the median SOX12 mRNA expression level. R packet Survival and Survminer conducted survival analysis using Kaplan-Meier curve. The Log-rank test was used to compare survival rates between different groups. P≤0.05 was considered statistically significant.

**Thyroid Differentiation Score (TDS) calculation**

TDS classifies samples based on the mRNA expression levels of 16 genes selected for thyroid function, including TG, TSHR, TPO, PAX8, FOXE1, SLC26A4, DIO1, DUOX2, NKX2-1, DIO2, GLIS3, SLC5A5 (NIS), THRA, THRB, DUOX1 and SLC5A8. Normalized RSEM log2 values were first clustered around the sample median to obtain log2 (fold change) and then summed over the 16 genes in each sample: TDS = mean of the 16 log2 genes (fold change).

**Immunohistochemistry (IHC)**

Tumor tissue sections were stained with the indicated antibodies using a standard immunohistochemistry protocol. The signal was visualized with the DAB Substrate Kit (ZLI-9017, Zsbio, China). The histoscore were calculated by two experienced pathologists as follows: histoscore = staining intensity × percentage of positive tumor cells. The staining intensity was regarded as 0 (no staining); 1 (weak, light yellow); 2 (moderate, light brown); or 3 (intense, brown). The percentage of positive cells was regarded as 0 (<5%), 1 (5–25%), 2 (25–50%), 3 (51–75%), or 4 (>75%).

**Transwell migration and invasion assay**

Cells were harvested and seeded on the upper layer of transwell chamber (Corning) at a density of 1.5 × 10^4^ cells per chamber for migration assay. The culture medium without fetal bovine serum was added for cell culture, while normal cell culture medium was added in the lower layer. After 24 hours of continued culture, the cells were fixed with paraformaldehyde and stained with 0.1% crystal violet. Three randomly selected fields of view were examined under a microscope and the stained cells were counted. For the invasion assay, cells were seeded on the upper layer of a Matrigel-coated transwell chamber and cultured for 48 hours.

**Wound healing assay**

Cells were plated in 6-well plates and grown until they reached confluence. Cell monolayers in 6-well plates was artificially scratched with 10 μL pipette tips. The wounded cell monolayer was washed and the wound areas were photographed under a microscope 0 and 12 h after scratching and measured using a caliper. Cell mobility was defined as the percentage of repaired area and calculated using the following formula: (1-(current wound size/initial wound size)) × 100.
